# Dot Immunobinding Assay Method with Chlorophyll Removal for the Detection of Southern Rice Black-Streaked Dwarf Virus

**DOI:** 10.3390/molecules17066886

**Published:** 2012-06-05

**Authors:** Zhuo Chen, Jiaju Liu, Mengjiao Zeng, Zhenchao Wang, Dandan Yu, Chengjun Yin, Linhong Jin, Song Yang, Baoan Song

**Affiliations:** 1 State Key Laboratory Breeding Base of Green Pesticide and Agricultural Bioengineering, Ministry of Education, Guizhou University, Guiyang 550025, China; 2 Key Laboratory of Green Pesticide and Agriculture Bioengineering, Ministry of Education, Guizhou University, Guiyang 550025, China

**Keywords:** Southern rice black-streaked dwarf virus (SRBSDV), dot immunobinding assay (DIBA), chlorophyll removal

## Abstract

Southern rice black-streaked dwarf virus (SRBSDV), a new virus from Fiji, has seriously damaged rice crops in southern China and northern Vietnam in recent years. This virus is difficult to diagnose in the early stages of infection, and is very destructive at the late stage. In the present study, a dot immunobinding assay (DIBA) that has a high sensitivity for diagnosing SRBSDV was developed. Two kinds of treatment for the DIBA were evaluated to determine the most effective one for removing chlorophyll interferences via rice extraction. The first included several reagents to remove chlorophyll, namely, the alkaline reagents like magnesium oxide and alumina oxide, the adsorbent reagents like activated carbon and bentonite, as well as the extraction agent acetone. The second and third treatments, which were used to remove chlorophyll in blot membrane-nitrocellulose and polyvinylidene fluoride (PVDF), included several organic solvents containing methanol, ethanol, acetone, ethyl acetate, and diethyl ether. The results showed that activated carbon and methanol yielded the best contrasting purple color for the infected samples by decreasing the chlorophyll content.

## 1. Introduction

Southern rice black-streaked dwarf virus (SRBSDV), which causes a malignant disease in rice, has become prevalent in recent years. This virus causes the destruction or death of rice, especially at the infection of the seeding stage [1,2]. SRBSDV has been detected in paddy fields covering nearly 1,193,333 ha in China in 2010, causing considerable damage in the provinces of Jiangxi, Hunan, Guangdong, Guangxi, Fujian, Hainan, Guizhou, Yunnan, and so on [3]. The rice disease caused by this virus has also been detected in northern Vietnam [4,5]. Previous studies have revealed that in the main regions of China, the disease was more serious in middle- and late-season rice crops than in early-season ones [3]. The viral disease caused by SRBSDV is latent (remains at the early infection stage) for a long time, and causes substantial damage at the late stage [2,3]. The disease it causes is difficult to prevent and control at the late stage, thereby it must be monitored and diagnosed at the early stage. Some studies have reported successful SRBSDV detection methods using reverse transcription-polymerase chain reaction (RT-PCR) and loop-mediated isothermal amplification (LAMP)-PCR against the gene of SRBSDV [2,4,5,6,7,8,9]. These laboratory methods are scientific and accurate, but rather laborious, time consuming, complicated, and expensive. Therefore, these methods are not readily available in rice cultivating areas, especially in rural villages.

The dot immunobinding assay (DIBA) is an enzyme-linked immunoassay based on blotting membranes [10]. The technique is similar to other DIBAs such as the dot enzyme-linked immunosorbent assay (ELISA) and nitrocellulose (NC)-ELISA [11,12]. The micro version of the technique is presently used to detect plant pathogens and some proteins from plant hosts because of its speed, simplicity of use, and minimal dependence on instruments [11,12,13,14,15]. In recent years, viral detection via their transmitting vectors has been extensively employed because this approach is fast, economical, and efficient [16,17]. One classical example is the detection of the rice strip virus via its vector, *Laodelphax striatellus* [18]. However, accurate results from DIBAs involving plant samples are difficult to obtain. False positive or false negative results are often obtained because of high levels of chlorophyll and low virus content in some plant tissue samples. Indeed, virus detection in an actual plant is less ideal than in a transmitting vector. Hence, the sensitivity of DIBA assays can be improved by removing chlorophyll or reducing its content in the plant tissue extract. Chlorophyll can be extracted and determined from plant tissues by a variety of established and relatively stable methods, such as grinding, filtering, and extraction [19,20]. However, few cases of chlorophyll removal for DIBAs have been reported. To remove chlorophyll from the blotting membranes in DIBA assays, Qian in a mini-review has demonstrated a method of washing the membranes for 30 min with 2% sodium hypochlorite before coloration [21]. Their study purpose is to wipe out the interference of chlorophyll before chromogenic reaction. Rocha-Peña has reported a method for decreasing green background using a blocking agent-3% gelatin treatment [22]. This method yields the best contrast between the green spots of chlorophyll and the purple spots of infected plants. In our previous study, two methods have been used to overcome chlorophyll interference. The first entails the improvement of the dilution-fold of rice extracts, and the second is the selection of plant tissues with low chlorophyll content (*i.e*., rice stem and root) [23]. However, these two methods alone do not change the chlorophyll content, and instead decrease the virus content. Hence, under the premise of ensuring the efficiency of antibody bonding to viral antigens, the current study was begun by systematically removing and reducing the chlorophyll levels. Among several chlorophyll removal techniques (*i.e*., reagent adsorption or destruction of chlorophyll, and washing of blotting membranes with organic solvents), two methods yielded the best contrast purple color for infected samples, indicating decreased chlorophyll content.

## 2. Results and Discussion

### 2.1. Chlorophyll Removal from Rice Extracts by Several Reagents

**Figure 1:**
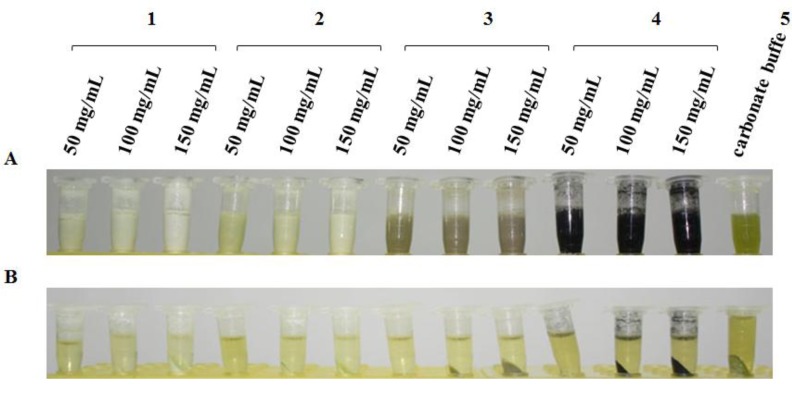
Effect of removing chlorophyll from each reagent treatment before and after centrifugation. (**A**) 1–4 represent magnesium oxide, alumina oxide, bentonite, and activated carbon (50, 100, and 150 mg/mL) added to the rice extract in carbonate buffer in Eppendorf tubes shaken for 3 min;5 is rice extract control in carbonate buffer without a reagent-removing chlorophyll. (**B**) 1–5 represent the same above listed treatment groups, which were centrifuged at 12,000 × *g* and 4 °C for 10 min.

As shown in Figure 1A, EPP tubes with rice extracts were centrifuged for 3 min at room temperature, then different contents of several reagents were added to the rice extract. The magnesium oxide (MgO) treatment group exhibited the best emulsifying effect on the carbonate-coated rice buffer, and did not present a clearly observable layer after several minutes. The alumina oxide (Al_2_O_3_) treatment group showed the second best emulsifying effect. Activated carbon had a dispersing effect, and the bentonite treatment displayed obvious precipitation after being left standing for several minutes. After the centrifugation of each treatment group at 12,000 × *g* and 4 °C for 10 min, the chlorophyll removed by several reagents was separated for precipitation. Chlorophyll in the precipitates was clearly observable on the MgO, Al_2_O_3_, and activated carbon groups. However, the bentonite treatment did not yield any obvious precipitation (Figure 1B).

**Figure 2:**
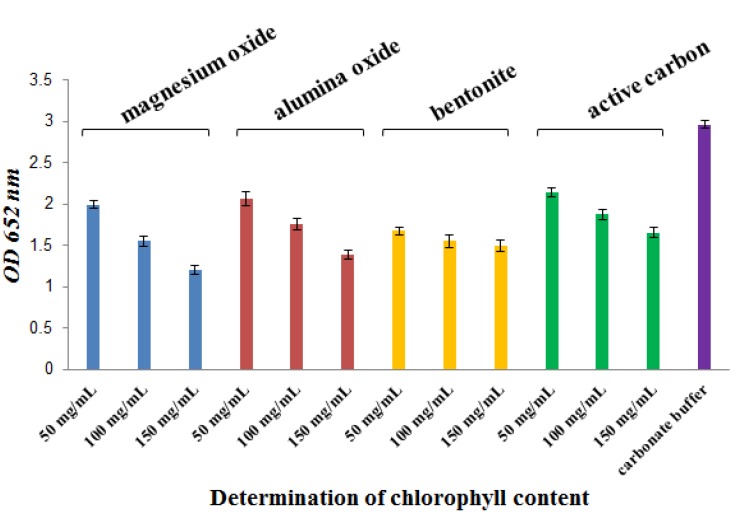
Determination of chlorophyll content in several reagents. Using the SPSS 11.5 software [24], data were statistically analyzed byANOVA (least significant difference). The results showed differences between the statistical data of the carbonate buffer treatmentand magnesium oxide, alumina oxide, bentonite, or activated carbon at 150 mg/mL (*p* < 0.05). There were statistically significant differences between the carbonate buffer treatment and magnesium oxide or bentonite at 50 and 100 mg/mL (*p* < 0.05). There were statistically significant differences in the same treatment group of magnesium oxide, alumina oxide, and activated carbon at 50 and 100 mg/mL (*p* < 0.05).

After centrifugation in several treatments, chlorophyll content was determined by the Arnon formula at 652 nm. As shown in Figure 2 and Table 1, chlorophyll content decreased the most in the Al_2_O_3_ and MgO treatment groups (*p* < 0.05, compared with the carbonate buffer group); chlorophyll decreased by 69.8% and 67.1%, respectively. A better result was shown in the bentonite treatment group. Chlorophyll content decreased the least in the activated carbon and Al_2_O_3_ treatment groups. Dependence on the content of added reagent was evident at the Al_2_O_3_, MgO, and activated carbon treatment groups (*p* < 0.05).

**Table 1:** Decreased chlorophyll content against several reagents.

Reagents	Concentration (mg/mL)	Decreased percent (%) ± SD
Magnesium oxide	50	67.1 ± 0.044
	100	52.3 ± 0.068
	150	40.7 ± 0.051
Alumina oxide	50	69.8 ± 0.087
	100	59.2 ± 0.069
	150	46.8 ± 0.052
Bentonite	50	56.5 ± 0.041
	100	52.2 ± 0.072
	150	50.7 ± 0.069
Active carbon	50	72.1 ± 0.052
	100	63.2 ± 0.064
	150	55.8 ± 0.06

Note: SD means standard deviation.

**Figure 3:**
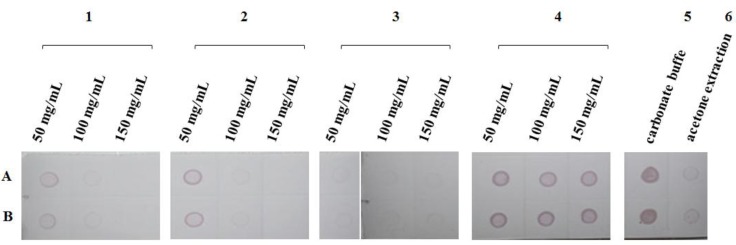
Color of NC membrane after DIBA processing with TBST and 5% non-fat dry milk for blocking as well as antibody and chromogenic detection of alkaline-phosphatase. Numbers 1–4 respectively represent magnesium oxide, alumina oxide, bentonite, and active carbon at 50, 100, and 150 mg/mL, respectively. Number 5 represents the rice extract in carbonate buffer, and number 6 represents the rice extract in buffer after acetone precipitation. Letters A and B represent two repeated samples. The rice infected by the SRBSDV was from the rice sample cultivated in the greenhouse.

As shown in Figure 3, when the rice extract treated with several reagents was used in a DIBA assay, the coloration effect in the dots around number 4 was better than in that around the other numbers. The MgO and Al_2_O_3_ treatments also displayed an appropriate coloration effect in terms of chlorophyll adsorption. The bentonite treatment possessed a strong capacity to absorb chlorophyll because of the absence of green spots. Via the analysis of various concentrations in the same treatment group, the MgO and Al_2_O_3_ treatment groups also exhibited appropriate adsorption capacity. In contrast, a small amount of chlorophyll was observed at 50 mg/mL, and chlorophyll content significantly decreased with increased treatment concentrations. No spot chlorophyll was observed at 150 mg/mL concentration. The lighter color may be related to the strong alkalinity of MgO and Al_2_O_3_, conferring an extremely destructive effect on the structural protein of the virus. In the bentonite treatment, no spot representing SRBSDV (hyacinthine) and chlorophyll (green) was observed at different concentrations. This finding indicated that bentonite induced the co-absorption of chlorophyll and the virus without selectivity. For activated carbon, adsorption was also observed, but was significantly weaker than for the other treatment groups. Moreover, although the rice extract solution of the carbonate buffer was added to eight times the volume of ice-cold acetone, the extraction effects of chlorophyll were evident. Chlorophyll dots did not occur, but numerous SRBSDV particles were lost in the entire experimental procedure.

### 2.2. Results of Chlorophyll Removal from NC Membranes by Washing with Several Organic Solvents

**Figure 4:**
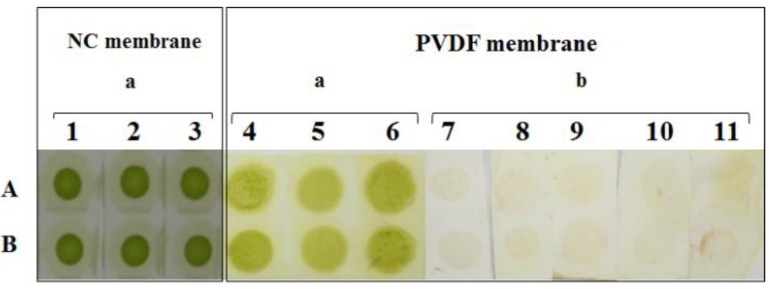
Effect of rice extract loading onto NC and PVDF membranes before and after washing. Numbers 1–3 represent the dry effect after loading the rice extract on the NC membrane. Numbers 4–6 represent the dry effect after loading the rice extract onto PVDF. Numbers 7–11 represent the washing effect of five kinds of organic solvent. A–B represent two repeats of the same experimental sample.

As shown in Figure 4, the effect of loading rice samples on the NC filter membrane was a green well-distributed spot. For the NC group treated with a variety of organic solvents, the elution effects of chlorophyll and the properties of each solvent-infected membrane were determined, as presented in Table 2. The sample in the NC membrane, washed by several organic solvents, did not exhibit an obvious chlorophyll-eluted effect. Nevertheless, the membrane was dissolved and damaged by some organic solvents, such as ethyl acetate and ether under certain concentrations listed in Table 2.

**Table 2:** Effect of chlorophyll removal and NC membrane destruction by organic solvents.

Organic solvent	Effect	Concentration					
		5%	10%	20%	30%	40%	50%
Methanol	Chlorophyll removal	Imperceptible	Imperceptible	Imperceptible	Slightly	Slightly	Slightly
	Membrane destruction	Imperceptible	Imperceptible	Imperceptible	Obvious	Obvious	Obvious
Ethanol	Chlorophyll removal	Imperceptible	Imperceptible	Imperceptible	Slightly	Slightly	Slightly
	Membrane destruction	Stable	Stable	Stable	Obvious	Obvious	obvious
Acetone	Chlorophyll removal	Imperceptible	Imperceptible	Imperceptible	Imperceptible	Imperceptible	Imperceptible
	Membrane destruction	Stable	Stable	Stable	Stable	Stable	Stable
Ethyl acetate	Chlorophyll removal	Imperceptible	Imperceptible	Slight	Slight	Slight	Slight
	Membrane destruction	Slight deformation	Obvious deformation	Slightly dissolved	Medium dissolved	Dissolved	Dissolved
Diethyl ether	Chlorophyll removal	Slight	Slight	Slight	Slightly	Slight	Slight
	Membrane destruction	Slight deformation	Slight deformation	Slight deformation	Slight deformation	Slight deformation	Slight deformation

### 2.3. Results of Chlorophyll Removal from PVDF Membranes by Washing with Several Organic Solvents

**Figure 5:**
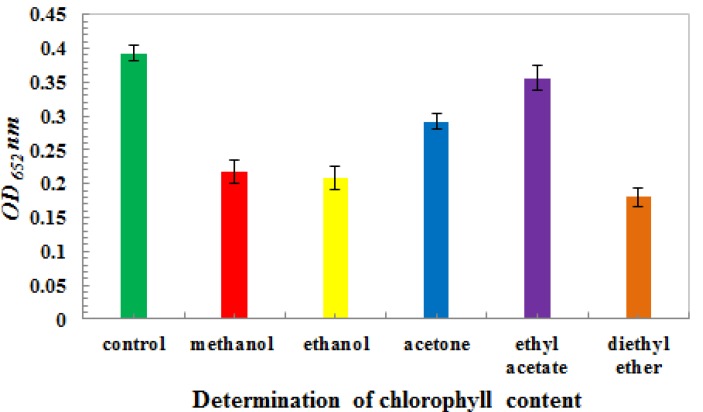
Determination of chlorophyll content in the PVDF membrane by washing with organic solvent. The chlorophyll content was presented as mean ± SD. Using SPSS 11.5, the data was statistically analyzed byANOVA (least significant difference). The results showed differences between the statistical data of the control and other treatment groups (*p* < 0.05), between methanol and ethyl acetate (*p* < 0.05),between ethanol and ethyl acetate (*p* < 0.05), between acetone and diethyl ether(*p* < 0.05), as well as between ethyl acetate and diethyl ether(*p* < 0.05).

As shown in Table 3, after PVDF dipped with methanol and rice extract sample loading, PVDF was washed with various organic solvents. As a result, better chlorophyll-eluting effects than in the NC membranes were observed. The PVDF structure was not destroyed by the organic solvents. The solution of each organic solvent used to treat the PVDF membranes had a temporary vortex. Chlorophyll content was detected by the Arnon method at a wavelength of 652 nm. As shown in Figure 5, the effect of chlorophyll removal from the PVDF membrane was very obvious (*p* < 0.05, between control and organic solvent treatment group).

**Table 3:** Effect of chlorophyll removal and PVDF membrane destruction by organic solvents.

Organic solvent	Effect	
Methanol	Chlorophyll removal	Obviously
	Membrane destruction	Stable
Ethanol	Chlorophyll removal	Obviously
	Membrane destruction	Stable
Acetone	Chlorophyll removal	Obviously
	Membrane destruction	Stable
Ethyl acetate	Chlorophyll removal	Obviously
	Membrane destruction	Stable
Diethyl ether	Chlorophyll removal	Obviously
	Membrane destruction	Stable

The decreased percentage of chlorophyll was calculated, as shown in Table 4. The elution effect in the ethyl acetate treatment group was the best among all treatments, with the percentage of decrease reaching 90.7%. The elution effect in the ether treatment group was the least desirable (51.0%). During chlorophyll removal, the PVDF membrane was washed with various organic solvents. The elution effects on chlorophyll were all ideal, but ether and ethyl acetate were found to be highly volatile, had pungent smell. Although acetone was also a good solvent for chlorophyll removal, it was also a protein denaturant and possibly undermined the antigen structure. Methanol had a good compatibility with the PVDF membrane as well, and is always used in the Western blot assay. Hence, methanol was chosen as the candidate solvent for washing the chlorophyll in the PVDF membrane, and was used in the DIBA test.

**Table 4:** Decreased percent of chlorophyll by organic solvent-induced membrane destruction.

	Concentration (%)	Reduced percent (%) ± SD
Methanol	100	54.1 ± 0.017
Ethanol	100	58.2 ± 0.018
Acetone	100	74.3 ± 0.011
Ethyl acetate	100	90.7 ± 0.018
Diethyl ether	100	51.0 ± 0.014

Note: SD means standard deviation.

**Figure 6:**
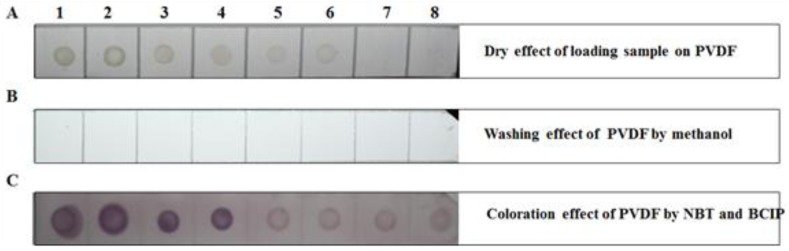
DIBA test completed in the PVDF membrane. (**A**) The dry effect of rice extraction in carbonate-coating buffer loading onto the PVDF membrane. (**B**) The effect of washing the membrane by methanol. (**C**) The coloration effect of the PVDF membrane with nitro-blue tetrazolium (NBT) and 5-bromo-4-chloro-3-inodlyl-phosphate (BCIP). Numbers 1 and 2 represent SRBSDV-infected rice in the greenhouse. Numbers 3 and 4 represent SRBSDV-infected rice acquired from Yuanjiang, Yunnan in 2011. Numbers 5 and 6 represent SRBSDV-infected rice acquired from Shidian, Yunnan in 2011. Numbers 7 and 8 represent SRBSDV-infected rice acquired from Dushang, Guizhou in 2010.

As shown in Figure 4 and Figure 6A, numbers 4–6 (Figure 4) and 1–4 (Figure 6A) had deep chlorophyll spots. The PVDF membrane was washed with methanol for tens of seconds until the majority of the chlorophyll was washed away (7–11 in Figure 4 and Figure 6B). The PVDF membrane was subjected to a DIBA test, and spots appeared in each treatment (Figure 6C), particularly for numbers 1–4. The dark-colored spot was ascribed to three molecular mechanisms. First, the site of recognition and combination to the first antibody was increased by the removal of chlorophyll. Second, the removal of chlorophyll decreased the interference of recognition between the first antibody and SRBSDV. Third and last, because of the green color limiting the visualization of the purple spot, the chlorophyll removal decreased the degree of green spotting of the blotting membrane. Consequently, the detection ratio against the SRBSDV was enhanced.

As shown in numbers 5–8 in Figure 6, the light-colored spot may be related to lower viral content. Hence, immunocapture (IC) RT-PCR assay of semi-quantity was verified against a positive control of SRBSDV-infected rice from the greenhouse at three dilution folds. As shown in Figure 7, two target products with a molecular weight of 877 and 427 bp about S5 and S10 gene in three differential dilution folds was obtained. The results further confirmed that a lower titer around SRBSDV with a light-colored spot was reasonable.

**Figure 7:**
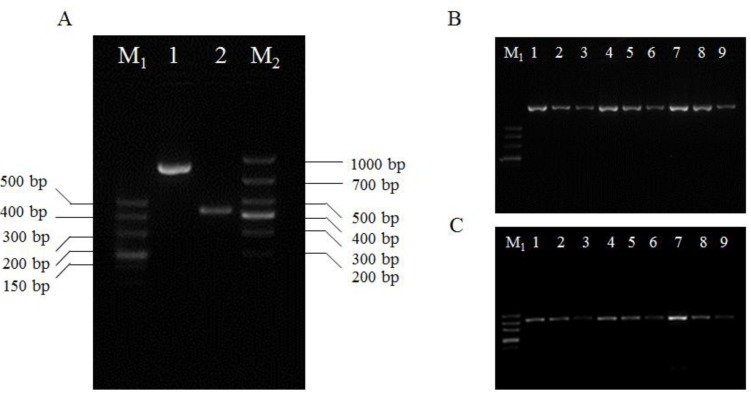
Demonstration test of IC-RT-PCR against a positive blot in the DIBA assay around the PVDF. “M_1_” and “M_2_” represent the DNA molecular weight marker (DL500 and DL1000 Marker, TaKaRa, Dalian). Part A: Lane 1 and 2 represent the gene product of S5 and S10. Part **B** and **C**: Lanes “1–3”, “4–6” and “7–9” represent the SRBSDV-infected rice from the greenhouse at three dilution folds: 10×, 100×, and 1,000×.

For the early diagnosis and monitoring of SRBSDV, methods for detecting nucleic acids in *Sogatella furcifera* and rice have been successfully developed [2,4,5,6,7,8,9]. However, for rapid detection in plant samples about serology method, high-content chlorophyll interference and low-content SRBSDV are obstacles yet to be overcome. Therefore, chlorophyll must be removed or decreased under the condition of invariant or increased SRBSDV content. Chlorophyll in rice is mainly grouped into chlorophyll a, chlorophyll b, carotene, and lutein; it is synthesized by a sequence of reactions beginning from glutamate with *δ*-aminolevulinic acid, porphobilinogen, uroporphyrinogen III, and the original chlorophyll acid. Chlorophyll is soluble in organic solvents (e.g., ethanol, methanol, and acetone) [25], and could be adsorbed by surface-active agents (e.g., activated carbon and diatomaceous earth) [26,27,28,29]. Hence, in the present study, several reagents containing MgO, Al_2_O_3_, and bentonite were evaluated for adsorption activity against chlorophyll, but not for viral particles. Activated carbon was determined as a good reagent among several reagents. To address the problem on solubility, chlorophyll-loaded NC and PVDF membranes were washed by organic solvents. Methanol was the best solvent for chlorophyll removal in terms of its non-destruction of the PVDF structure, volatility, and low toxicity to humans. The SRBSDV content in the sample was very low, whereas the chlorophyll content was extremely high, such as in the seeding stage. The present study also adopted a method suitable for extremely concentrated viruses, involving the pre-cooling of acetone for extraction. The pre-cooled acetone denatured and precipitated the viral proteins, thereby concentrating the virus. However, the complicated operation and easily lost viral proteins rendered the method unsuitable for the rapid detection of the SRBSDV. Furthermore, although 2% sodium hypochlorite solution has been reported to eliminate the chlorophyll blot on membranes, this reagent was mainly used in the coloration process in the present study. The 3% gelatin can eliminate the background caused by the green spot, but cannot decrease chlorophyll adsorption in the blotted membrane [21,22]. Therefore, the best way to remove chlorophyll is to have it adsorbed by activated carbon. The sample should then be loaded on a PVDF membrane, and the PVDF membrane should be washed with methanol. Both methods have ideal effects on chlorophyll removal, and can thus be adopted when treating samples with high chlorophyll contents.

## 3. Experimental

### 3.1. Materials

The SRBSDV S10 polyclonal antibody was prepared according to the polypeptide method previously used by our group [23]. Polypeptide information was obtained using a sequence specified by bioinformatics. The polypeptide was synthesized by peptide synthesis technology. The sequence of the polypeptide was CRNDQPTRNTNLSLSQSTENR. Polyclonal antibody S10 was prepared according to the conventional approach. Its titer and specificity were evaluated by Western blot assay and indirect ELISA [23]. The second antibody was goat-anti-rabbit second antibody marked by alkaline phosphatase, purchased from the Doctor Bioengineering Co., Ltd. (Wuhan, China). Nitroblue tetrazolium (NBT; Sigma) and 5-bromo-4-chloro-3-inodlyl-phosphate (BCIP; Sigma) were purchased from the Solarbio Technology Co., Ltd. (Beijing, China). The NC membrane (0.22 μm), PVDF membrane (0.22 μm), Prime Script® One Step RT-PCR Kit Ver. 2 (DRR055A), and DL500 DNA Marker were from the PALL Company (New York, USA), Millipore Company (Billerica, MA, USA), and TaKaRa Ltd. (Dalian, China), respectively. MgO, Al_2_O_3_, bentonite, and acetone were analytical grade. The activated carbon (8–16 mesh) used for adsorption was obtained from the Aladdin Company. The primer sequences of SRBSDV S10 gene was designed by the Primer 5 software according to its sequence information (National Center for Biotechnology Information reference sequence NC_014713.1) in GenBank. The sequences of the left and right primers of the S5 and S10 gene were 5'-agattctgtcagtgattacgtagtt -3' and 5'-tgtgactgagccagtgaagg -3', 5'-gaacaaacatggagcggagt -3' and 5'-atgccttaccacgtttccag -3', respectively. The primer synthesis was conducted in TaKaRa Ltd. The rice materials used in the DIBA and IC-RT-PCR are shown in Table 5. S5 and S10 gene sequencing and sequence alignment were conducted, and SRBSDV was found to be very conservative and nonexistent in virus strains. The product was expected to be 877 and 427 bp long.

**Table 5:** Information on the rice materials used in the experiment.

Sample source	Rice cultivars	Sowing date	Year
Indoor growing, artificially infected by *Sogatella furcifera*	Indica type rice		2011
Paddy field, collected from Yuanjiang county, Yunnan province	Indica hybrid rice	Early season rice	2011
Paddy field, collected from Shidian county, Yunnan province	Indica hybrid rice	Early season rice	2011
Paddy field, collected from Dushan county, Guizhou province	Indica type rice	Single harvest rice	2010

### 3.2. Methods

#### 3.2.1. Preparation of Rice Extract

About 2 g of fresh rice leaves infected by SRBSDV was weighed and ground into a powder in liquid nitrogen. After the liquid nitrogen volatilized, the sample was coated with 0.05 mol/L carbonate buffer (containing 0.015 M Na_2_CO_3_ and 0.035 M NaHCO_3_) at a ratio of 1:3 (g/mL). The homogenate was continuously ground until the particles and sediments became invisible. The ground sample was then transferred with 1 mL of lapping fluid into a 1.5 mL Eppendorf (EPP) tube.

#### 3.2.2. Treatment Methods for Eliminating Chlorophyll

For sample treatment (**A**), 1 mL of lapping fluid was transferred into a 1.5 mL EPP tube, which was centrifuged at 12,000 × *g* at 4 °C for 10 min. The supernatant was transferred into a new EPP tube, and directly loaded onto the NC membrane. For the MgO treatment (**B**), 50 mg of MgO was placed in an EPP tube, which was shaken and blended for 3 min. For the Al_2_O_3_ treatment (**C**), 50 mg of Al_2_O_3_ was placed in a 1.5 mL EPP tube, which was shaken and blended for 3 min. For the bentonite treatment (**D**): 50 mg of bentonite was placed in a 1.5 mL EPP tube, which was shaken and blended for 3 min. For activated carbon treatment (**E**), the bulk activated carbon was first ground into powder, and 50 mg of activated carbon powder was placed in a 1.5 mL EPP tube. The tube was shaken and blended for 3 min. Numbers 2–5 were processed by a variety of treatments and centrifugation at 12,000 × *g* and 4 °C for 10 min. The supernatant was transferred into a new EPP tube and stored at −20 °C for later use. For the acetone treatment (**F**), the material was homogenized by grinding the sample solution and adding eight times the volume of ice-cold acetone. The mixture was placed in a −20 °C refrigerator for 15 min to promote protein precipitation and liquid acetone removal. Centrifugation at 12,000 × *g* and 4 °C for 10 min was performed, the supernatant was discarded, and the sediments were set aside. After the precipitate dried, the original volume of the coated carbonate buffer was added to promote protein dissolution. The supernatant was then obtained via instantaneous centrifugation. For the blot treatment (**G**), 5 μL of extracted carbonate buffer was coated onto the NC and PVDF membranes. Until the membranes dried and with every two sample points as a treatment group, the NC and PVDF membranes were washed for 30 s in 1 mL each of methanol, ethanol, acetone, ethyl acetate, and ethyl ether solutions with different concentrations. Washing was repeated 20 times for each treatment. The details are shown in Figure 7 as well as Table 1 and Table 2.

#### 3.2.3. DIBA Test

About 5 μL of the sample solution was loaded onto the NC and PVDF membranes, which were naturally volatilized). After membrane drying, the blot membrane was placed in 5% non-fat milk and TBST buffer placed in a water bath at 37 °C for 30 min. The TBST buffer contained 8.8 g of NaCl and 20 mL of 1 M Tris-HCl (pH 8.0) dissolved in 800 mL of ddH_2_O, and mixed with 0.5 mL of Tween-20. The final volume was 1,000 mL, and the solution was stored at 4 °C. Subsequently, a 1:1,500 first antibody solution (with 5% non-fat milk-TBST) was incubated for 60 min at 37 °C. The blot membrane was thrice washed with TBST for 3 min each time. Up to 15 mL of AP-conjugated goat anti-rabbit antibody solution (with 5% non-fat milk-TBST) was incubated for 40 min at 37 °C. The membrane was thrice washed for 3 min each time. The NC and PVDF membranes were then mixed with a chromogenic substrate buffer (containing 0.1 M Tris, 0.1 M NaCl, and 0.05 M MgCl_2_·6H_2_O, pH 9.5) for 2 min. About 15 μL of 50 mg/mL NBT (50 mg of NBT dissolved in 1 mL of *N*,*N*-dimethylformamide, and mixed in a vortex mixer for 3 min) was mixed with 10 mL of substrate buffer solution. About 25 μL of 50 mg/mL BCIP (containing 50 mg of BCIP dissolved in 1 mL of *N*, *N*-dimethylformamide, and mixed in a vortex mixer for 3 min) was also added dropwise with gentle mixing into the NBT solution. The coloration was terminated by adding ddH_2_O when the color became clear. The period of coloration was determined by the time at which positive control spots appeared. The appearance of negative control spots was not significant for the termination of coloration.

**Figure 7:**
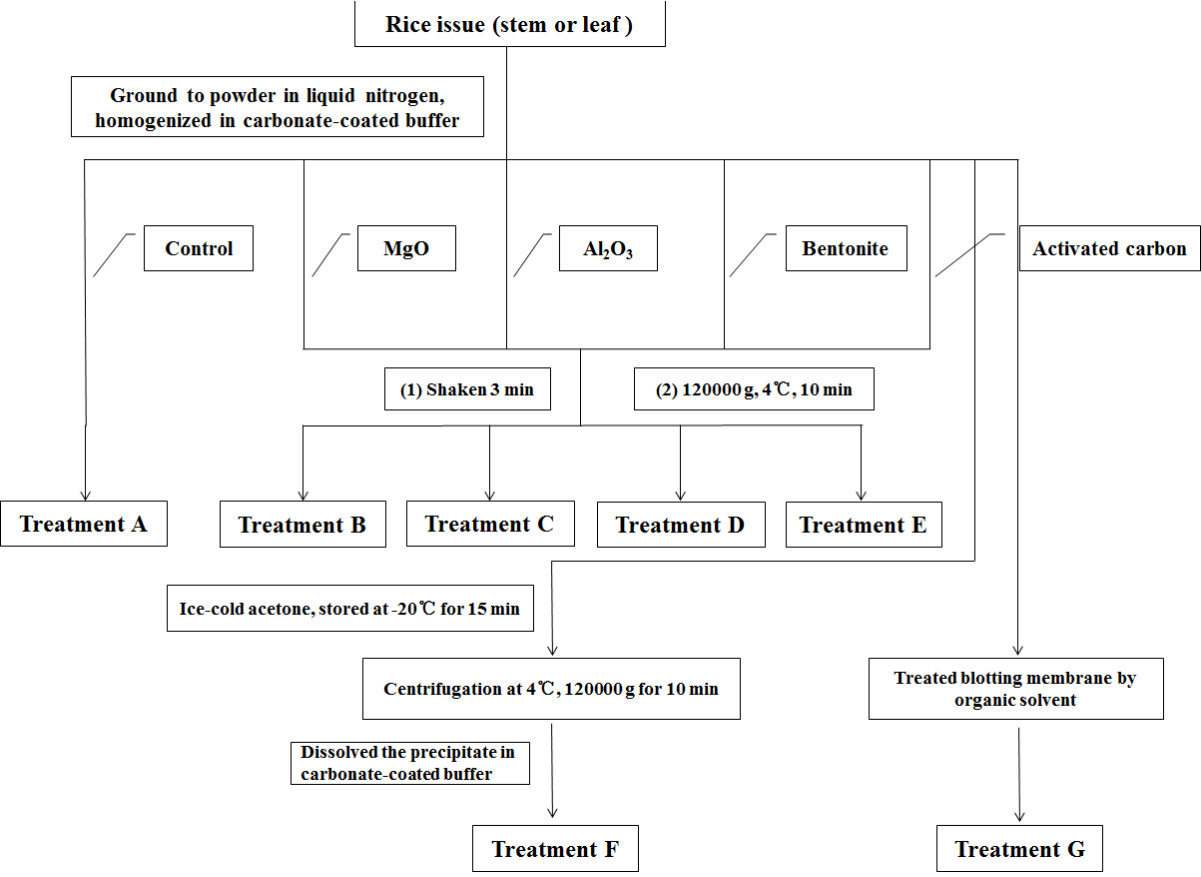
Treatments for removing chlorophyll from rice extract solutions.

#### 3.2.4. IC-RT-PCR Demonstration Test

Referring to the method of Ouyang [17], the IC-RT-PCR assay was conducted with slight modifications. A sample of SRBSDV-infected rice from the greenhouse was subjected to IC-RT-PCR at 10-, 100-, and 1000-fold dilutions. About 200 μL of 1:1,500 antibody solutions (diluted with PBST buffer) was poured into PCR tubes and incubated for 60 min at 37 °C. The antibody solution was discarded, and 200 μL of differently diluted rice extracts was incubated at 37 °C for 60 min. The sample solution was discarded, thrice washed with PBST buffer, and once washed with RNase-free diethylpyrocarbonate water. A one-step PCR reaction was used at the following conditions: 50 °C for 30 min, 94 °C for 2 min, 94 °C for 30 s, 58 °C for 30 s, and 30 cycles at 72 °C for 50 s. Agarose gel (2.5%) was used for electrophoresis under a 100 V constant voltage. The samples were stained by electron beam and ultraviolet (UV) imaging to observe the PCR products.

#### 3.2.5. Determination of Chlorophyll Content

Several treatment groups in Section 3.2.2 were measured for chlorophyll content using a UV spectrophotometer. For the assays of treatments A–E and G, treatment group A was set as the control group in the A–E assay. A control treatment of pure grinding fluid solution in methanol (15 μL of grinding supernatant directly added to methanol to determine chlorophyll content) was set for the G assay. The Arnon formula was applied at a wavelength of 652 nm, as follows [19]:


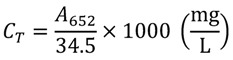



The percent decrease of chlorophyll was calculated as follows:


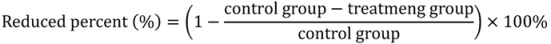



#### 3.2.6. Statistical Analysis

SPSS 11.5 was used for the statistical analyses of the data on measured chlorophyll content. ANOVA (least significant difference method) was performed to analyze the differences among the treatment groups [24].

## 4. Conclusions

In the present study, a dot immunobinding assay (DIBA) for removing chlorophyll that has a high sensitivity for diagnosing the SRBSDV was developed. Three kinds of treatment for the DIBA were evaluated to remove chlorophyll interference via rice extraction. These treatments include alkaline reagents—magnesium oxide and alumina oxide, adsorbent reagents—activated carbon and bentonite, extraction agent—acetone, and blot membrane-PVDF and NC membrane effect on chlorophyll removal. The results showed that removing chlorophyll by activated carbon and washing PVDF membrane with methanol gave the best contrasting purple color for infected samples through decreasing chlorophyll content. The method has been successfully applied to a dot immunobinding assay (DIBA) that has the most effective technique for diagnosing the SRBSDV in rice.
